# Visual and textual cues in online presentations of natural foods are associated with taste inference and cognitive engagement

**DOI:** 10.1371/journal.pone.0351115

**Published:** 2026-06-18

**Authors:** Lili Sun, Chenwen Wei, Heliang Huang

**Affiliations:** 1 College of Economics and Management, Fujian Agriculture and Forestry University, Fuzhou, Fujian, China; 2 School of Economics and Management, Xidian University, Xi’an, Shaanxi‌‌, China; Universidad Nacional de Tres de Febrero, ARGENTINA

## Abstract

Digital environments have become important contexts in which consumers form sensory expectations and evaluate food quality prior to consumption. Drawing on the elaboration likelihood model and attribution theory, this study develops a theoretically grounded process model to explain how visual and textual cues in online presentations of natural foods shape food-related cognition. Specifically, we propose that perceived naturalness serves as an initial perceptual input that can trigger cognitive engagement through multiple mechanisms: directly, via credibility as a validation mechanism, via taste inference as an experiential simulation, and through a sequential chain in which credibility enables taste inference that subsequently sustains elaboration. A 2 (platform type: content-oriented vs. transaction-oriented) × 2 (image scene: lifestyle-oriented vs. nature-oriented) × 2 (text framing: consumption-oriented vs. production-oriented) between-subjects experiment (N = 320) was conducted. Partial least squares structural equation modeling was employed to test direct and indirect effects; multi-group analysis examined boundary conditions across experimental contexts; and necessary condition analysis identified minimum required levels of predictors for high engagement states. The results indicate that perceived naturalness has a significant direct effect on cognitive engagement, as well as indirect effects through credibility and taste inference independently and in sequence. The indirect pathway is more pronounced in content-oriented environments, particularly when nature-oriented images and consumption-oriented text are used. Taste inference emerged as the strongest necessary condition for high cognitive engagement, followed by credibility; perceived naturalness showed a weaker but significant necessity effect. These findings demonstrate how visual and textual cues jointly guide anticipatory sensory processing and cognitive engagement in digital food contexts, offering both theoretical contributions to cue-based processing research and practical implications for the design of online presentations of natural foods.

## Introduction

Consumers increasingly evaluate foods prior to consumption through digital representations rather than direct sensory experience. In online environments, product covers and home-feed cards—typically combining images with brief textual descriptions—often serve as primary interfaces through which consumers form expectations about food quality, freshness, and taste. These early impressions shape food-related cognition, particularly for natural foods, whose value is closely linked to perceptions of purity, origin, and authenticity. On product covers, visual elements evoke rapid impressions of attributes such as naturalness or freshness, whereas textual framings provide semantic information about origin and production processes, supporting more deliberate interpretation [1,2]. Together, these cues influence how consumers interpret food-related information and engage in subsequent cognitive processing [3].

Within this context, perceived naturalness represents a particularly influential evaluative dimension. Labels and imagery emphasizing attributes such as “additive-free”, “organic”, or “natural” address food safety concerns while also aligning with consumers’ lifestyle values [4,5]. Prior research indicates that perceived naturalness is associated with food evaluation, trust, and consumption-related judgments [6,7]. Yet existing evidence offers limited insight into how cover-level visual and textual cues jointly shape naturalness perceptions—and, more importantly, how these perceptions translate into deeper food-related cognition prior to consumption.

One way naturalness may operate is by shaping consumers’ assessment of whether presented information can be relied upon. Drawing upon attribution theory [8], consumers actively attribute meaning to naturalness cues by evaluating their diagnosticity in signaling underlying product attributes [9]. When naturalness cues are perceived as diagnostic, they trigger positive attribute inferences [10], which subsequently influence credibility. Foods presented through cues signaling purity or minimal processing are more likely to be regarded as authentic and trustworthy [4]. In digital environments, credibility functions as an important psychological link between external cues and internal evaluations, influencing the extent to which consumers rely on and elaborate upon food-related information [11,12].

But credibility is not the only mechanism at play. When food information is perceived as credible, consumers are more likely to engage in mental simulation, such as imagining taste, texture, or freshness [13,14]. Drawing upon the elaboration likelihood model (ELM) [15], high source credibility may enhance consumers’ motivation to engage in central route processing, allowing them to more carefully evaluate product-related information and form more accurate taste expectations [16,17]. This process, referred to as taste inference, represents a cognitive mechanism through which consumers predict sensory attributes based on available visual and textual cues [18,19]. Common features of natural food imagery, including unspoiled landscapes and cues of minimal processing, activate sensory representations associated with freshness and natural flavor [20,21], facilitating anticipatory sensory processing [14,22]. Importantly, consumers can also form taste inferences directly from naturalness cues when sensory imagery is vivid and self-relevant [23], without necessarily passing through credibility judgments first.

These anticipatory sensory judgments, in turn, may sustain consumers’ cognitive investment. In contemporary digital environments, food evaluation involves active cognitive engagement—developing interest in the product, seeking detailed information, and exploring additional content [24]. These efforts reflect deeper levels of information processing as described in the ELM. Taste inference provides an informational basis that motivates consumers to move from initial cue-based evaluation toward more elaborative, central-route processing [25,26]. Yet naturalness itself may also compel such engagement directly, by capturing attention and signaling product value worth exploring [6,27]. The relative contributions of these direct and indirect pathways remain unclear.

Theoretically, perceived naturalness may influence cognitive engagement through multiple pathways: directly, via credibility as a validation mechanism, via taste inference as an experiential simulation, and a sequential chain from credibility to taste inference. These pathways collectively sustain elaboration. However, these possibilities have not been examined within a single empirical framework, leaving uncertainty about how naturalness cues operate in digital food contexts.

This uncertainty is compounded by the role of context. Digital platforms differ fundamentally in the experiences they afford. Content-oriented platforms emphasize experiential sharing and community interaction, whereas transaction-oriented platforms prioritize utilitarian information and purchase efficiency, potentially guiding different modes of food-related processing [28,29]. Within a given platform, cover image scenes may evoke different attributions—natural landscapes signaling authenticity versus lifestyle contexts signaling convenience [30,31]. Textual framings may activate different motivations, with consumption-oriented language emphasizing self-relevant experience and production-oriented language emphasizing external verification [18,32]. How these contextual factors shape the pathways from naturalness to engagement remains poorly understood.

To address these gaps, the present study draws on the ELM and attribution theory to examine how consumers evaluate natural foods in digital environments [8,15]. Attribution theory explains how consumers evaluate the diagnosticity and credibility of naturalness cues, while the ELM explains how such evaluations shape the depth of subsequent cognitive processing. Integrating these perspectives, we propose that perceived naturalness serves as a perceptual input that can trigger cognitive engagement through multiple mechanisms. Credibility functions as a validation mechanism that reduces uncertainty and fosters elaboration. Taste inference provides an experiential simulation that sustains motivation for deeper processing. These pathways may operate independently or in sequence, where credibility enables taste inference, creating a serial pathway from cue validation to sustained elaboration. This framework captures both parallel and sequential dynamics in how naturalness cues translate into cognitive engagement.

Accordingly, this study addresses the following research questions:

1. How do perceived naturalness, credibility, and taste inference collectively influence cognitive engagement—through direct effects, independent mediation, serial mediation, or a combination of these?
2. Do platform type, cover image scene, and textual framing influence the strength of these associations?
3. Among perceived naturalness, credibility, and taste inference, which factors are critical for triggering meaningful levels of cognitive engagement?

By addressing these questions, the study provides empirical evidence on how visual and textual representations of natural foods shape anticipatory sensory judgments and cognitive engagement in digital contexts, offering practical insights for the design of online food presentations.

## Theoretical background and hypotheses

### Theoretical foundation: ELM and attribution theory

When consumers browse natural food products online, they face a fundamental problem: they cannot touch, smell, or taste the food before purchase. They must decide, based solely on digital cues, whether a product claiming to be “natural” truly warrants their cognitive investment—or whether it is merely another marketing gloss. This study integrates the elaboration likelihood model (ELM) and attribution theory to explain how consumers navigate this uncertainty.

ELM posits that persuasive information is processed through two routes [15]. The peripheral route relies on heuristic cues for quick judgments, while the central route involves effortful evaluation producing enduring attitudes [33]. Motivation and ability determine which route dominates [34]. Yet ELM remains silent on a critical question: what makes consumers believe a peripheral cue is worth the effort of central-route processing? Attribution theory fills this gap by explaining how individuals infer causes from observable effects and judge information validity under uncertainty [8]. In digital food contexts, consumers must determine whether naturalness cues reflect genuine product attributes or promotional tactics—an authenticity attribution that decides whether deeper elaboration is warranted [35]. Together, these theories capture both the process of cognitive deepening (ELM) and the mechanism of cue validation (attribution theory) that drives it.

### The direct path: When naturalness compels immediate attention

Perceived naturalness serves as the primary evaluative dimension during the initial encounter with food imagery—how authentic, minimally processed, and close to nature the product appears [6,7]. This rapid perceptual appraisal emerges from visual-textual cue integration, operating as the initial peripheral trigger that captures attention and sets subsequent processing in motion [1,36].

But does this initial impression stop at superficial liking, or can it directly fuel deeper engagement? We argue for the latter. Consumers associate naturalness with health, minimal processing, and quality [6]. When cover images present natural, unretouched features, they implicitly communicate origin, production process, and purity—information that stimulates active exploration [37]. Visual naturalness acts as an efficient cognitive trigger, lowering cognitive defense and encouraging systematic evaluation of product value [27]. This sustained mental investment—encompassing interest in the product, intent to acquire detailed information, and willingness to explore additional content—constitutes cognitive engagement [38,39], the ultimate outcome we seek to explain. Based on this, we propose:

**H 1** *The higher the perceived naturalness of the e-commerce product card cover, the stronger consumers’ cognitive engagement.*

### The credibility route: How trust mediates the naturalness effect

Yet not all consumers who encounter natural cues will engage deeply. Some may dismiss them as mere decoration. What determines whether naturalness translates into engagement? One path runs through consumers’ assessment of whether the presented information can be relied upon—whether the naturalness they perceive reflects genuine product attributes or marketing manipulation. This meta-evaluative judgment of trustworthiness and validity, which we call credibility [11], emerges from causal attribution and operates at an intermediate level of abstraction between initial perception and deeper response.

In e-commerce, consumers cannot verify quality directly, so they rely on observable cues to compensate for virtual uncertainty [40]. Natural-style images evoke perceptions of minimally processed, naturally sourced products [41], which—when aligned with prior experience—are interpreted as evidence of actual attributes rather than promotional tactics [35,42]. This attribution of authenticity reduces skepticism and increases perceived credibility [43]. Within ELM, credibility functions beyond superficial processing: it generates trust that lowers psychological resistance, creating conditions for central-route elaboration [15,44]. Consumers who find natural cues credible become willing to explore origin, production methods, and health value—behaviors that manifest as heightened cognitive engagement [38]. Based on this, we propose:

**H 2** *Credibility mediates the positive effect of perceived naturalness on cognitive engagement*.

### The sensory route: Inferring taste from perceived naturalness

Credibility explains the rational path to engagement, but naturalness may also operate through a more experiential channel. When consumers encounter natural food imagery, they often find themselves imagining how the product would taste—its freshness, texture, and flavor. These anticipatory sensory expectations, built from stored memories and activated by visual cues, constitute taste inference [18,19]. Drawing on the elaborated intrusion theory of desire [45], such mental simulations sustain motivation and amplify the affective pull of food information, transforming cue-based evaluation into goal-directed processing [23,46].

Images of pristine landscapes or unprocessed features evoke imagined sensory experiences—freshness, naturalness, original flavor—that promote vivid mental simulation [14,20,22]. Importantly, this simulation is not passive entertainment; it motivates active exploration such as checking details, reading reviews, and verifying origins [44]. Taste inference thus serves as a bridge linking the sensory promise of naturalness to the behavioral investment of engagement [47]. Based on this, we propose:

**H 3** *Taste inference mediates the positive effect of perceived naturalness on cognitive engagement.*

### The serial mediation paths: From initial perception to cognitive elaboration

Credibility and taste inference not only operate independently but also form a sequential pattern: consumers first validate naturalness cues as trustworthy, then allow that validated information to fuel sensory imagination, which finally drives sustained cognitive engagement. This ordering reflects a hierarchy of cognitive abstraction consistent with both ELM and attribution theory.

In this sequential pattern, credibility validates perceptual input as diagnostic rather than deceptive [8,48]. Taste inference, in turn, benefits from this validation—sensory simulation proceeds more freely when uncertainty about cue validity is reduced [17,49]. Empirically, credibility enhances willingness to rely on product information, thereby facilitating sensory inference [11,44]. Once taste inference generates experiential motivation, consumers engage in the exploratory behaviors that constitute cognitive engagement [50,51]. This sequential pattern complements the parallel mediating roles of credibility and taste inference by highlighting the synergistic role of validation and simulation in driving engagement. Based on this, we propose:

**H 4** *Credibility and taste inference sequentially mediate the positive effect of perceived naturalness on cognitive engagement.*

### When context alters the process‌‌

The effect of perceived naturalness on cognitive engagement is unlikely to be uniform. The same natural food image may prompt deep elaboration in one setting yet be dismissed as decoration in another. This variability depends on the environment in which cues are encountered. We examine three contextual factors that alter this process, each operating at a distinct stage of the elaboration sequence.

#### Platform type: Community or marketplace?.

Digital platforms differ fundamentally in the experiences they cultivate. Content-oriented platforms, such as Xiaohongshu and Douyin, immerse users in emotional resonance and lifestyle narratives, emphasizing community interaction and experience sharing [28,52]. In these environments, consumers interpret naturalness cues as reflections of genuine user experience rather than marketing efforts, enabling mental simulation and deep cognition [53]. Transaction-oriented platforms such as Taobao and Pinduoduo emphasize efficiency and purchase completion [29]; here, consumers attribute naturalness to promotional tactics, conserving cognitive resources for heuristic cues like discounts [54].

Critically, this platform distinction shapes how credibility assessments translate into sustained cognitive engagement. On content-oriented platforms, the emphasis on community interaction and experience sharing [28,52] validates exploration behavior, strengthening the link between credibility beliefs and elaborative processing. On transaction-oriented platforms, the focus on efficiency and purchase completion [29] leads consumers to prioritize heuristic cues like discounts over deep elaboration [54], attenuating this downstream translation. Based on this, we propose:

**H 5** *Platform type moderates the effect of perceived credibility on cognitive engagement, with a stronger positive relationship on content-oriented than transaction-oriented platforms.*

#### Cover image scene: Origin or usage?.

The visual story told by the image matters. Natural scenes—origin landscapes, pastoral settings—reinforce the product-environment connection, prompting consumers to attribute naturalness to true origin and production process [30]. This mental mapping activates authenticity expectations that drive central processing [55]. Lifestyle scenes—dining tables, home contexts—convey convenience and emotional warmth but highlight usage rather than authenticity [31]. They resonate affectively yet do not invite exploration of product value, remaining at the heuristic level [1,56].

Moreover, natural scenes may establish a direct channel from visual naturalness to cognitive engagement, bypassing inferential processing. When the image itself embodies the naturalness claim—through origin landscapes and pastoral settings that reinforce the product-environment connection [30]—consumers need not rely on credibility assessments to justify their engagement; the visual evidence is self-validating, activating authenticity expectations that drive central processing [55]. Lifestyle scenes—dining tables, home contexts—convey convenience and emotional warmth but highlight usage rather than authenticity [31]. They resonate affectively yet do not invite exploration of product value, remaining at the heuristic level [1,56], making engagement contingent on prior credibility formation. Based on this, we propose:

**H 6** *Cover image scene moderates the effect of perceived naturalness on (a) perceived credibility and (b) cognitive engagement, with stronger effects for natural than lifestyle scenes.*

#### Cover textual framing: Experience or evidence?.

Finally, the words accompanying the image shape how naturalness is processed. Consumption-oriented framing emphasizes usage experience, benefits, and rewards, activating self-relevance motivation that encourages central-route processing [18]. Consumers link naturalness to personal experience value, forming taste inferences that deepen engagement. Production-oriented framing emphasizes origin and supply chain, providing external verification that enhances intuitive authenticity judgment but does not trigger the experiential motivation necessary for deep elaboration [32]. Its persuasive power depends on external credibility rather than internally driven processing.

Importantly, this framing distinction operates at the intermediate stage between credibility and taste inference. Consumption-oriented language functions as an experiential prime, prompting consumers to translate their credibility assessments into sensory simulations. Production-oriented language, while bolstering credibility, does not provide the experiential scaffold needed to activate taste imagination. Thus, textual framing governs whether credibility beliefs cascade into inferential processing. Based on this, we propose:

**H 7** *Textual communication framing moderates the effect of perceived credibility on taste inference, with a stronger positive relationship under consumption-oriented than production-oriented framing.*

**Fig 1:**
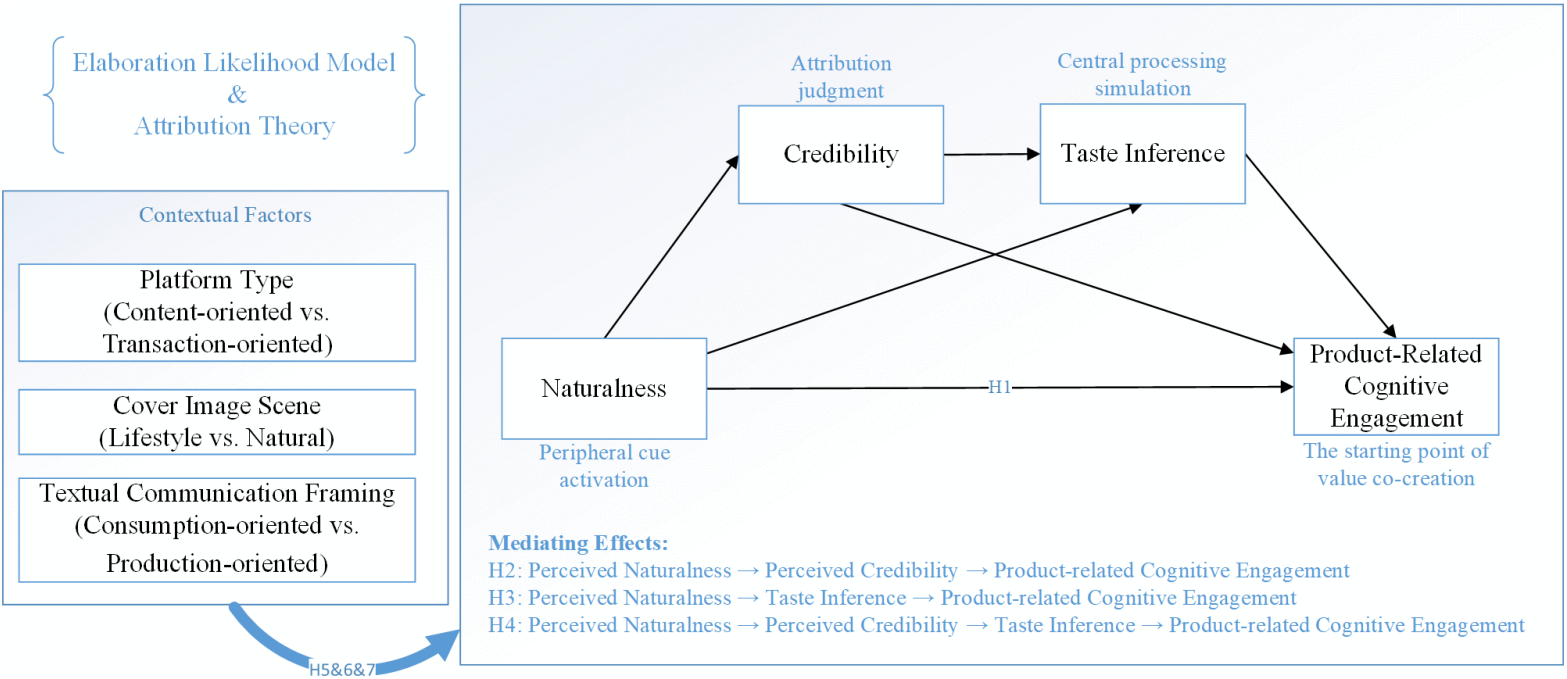
The conceptual model.

Based on the above theories and hypotheses, the proposed conceptual model is shown in Fig 1.

## Materials and methods

### Participants and design

#### Ethics statement.

This study was exempt from ethical review. The research involved an anonymous online survey with no sensitive personal information or invasive procedures and posed minimal risk to participants. According to Article 32 of the Regulations on Ethical Review of Life Science and Medical Research Involving Humans issued by the National Health Commission of China (Document No. [2023] 21), such low-risk anonymized research is exempt from ethical review. All participants provided electronic informed consent prior to participation. The consent process included: (a) a brief description of the study purpose, procedures, anonymity protections, data usage, and right to withdraw; (b) an explicit confirmation question (“Have you read and understood the informed consent and voluntarily agree to participate in this study?”); and (c) participants’ affirmative response and proceeding to the survey as recorded consent. Participants could withdraw at any time without penalty by closing the browser.

#### Design.

The study employed a 2 (platform type: content-oriented vs. transaction-oriented) × 2 (cover image scene: natural vs. lifestyle) × 2 (textual communication framing: consumption-oriented vs. production-oriented) between-subjects factorial design. Perceived naturalness was the independent variable, perceived credibility and taste inference were measured as mediators, and product-related cognitive engagement was the dependent variable.

#### Participants and recruitment.

Participants were recruited via Credamo from June 21 to July 7, 2026. Credamo is a professional Chinese online research platform recognized for its robust participant pool and stringent quality control mechanisms. A total of 360 invitations were sent. To ensure high data quality, a multi-stage screening process was implemented. First, we embedded Instructional Manipulation Checks (IMC): participants were required to select a specific response on a 7-point scale (e.g., “Select ‘1’ for this item”) and correctly identify a logic-based anchor (e.g., identifying “Teacher” as the profession responsible for school instruction). A total of 32 responses were automatically rejected by the platform’s system due to failed IMCs, detected data anomalies, or excessive full-screen exits (indicating potential distractions). Second, demographic cross-verification was conducted by matching self-reported birth years with selected age groups; inconsistent responses were discarded. Finally, we applied response time filtering, manually excluding 8 additional participants who completed the survey in less than 90 seconds or more than 1,200 seconds. This process yielded a final sample of 320 valid participants (valid response rate = 88.9%). This sample is specific to the Chinese e-commerce context and is not intended to represent Western or other cultural markets.

A priori power analysis was conducted using G*Power 3.1 [57]. For multiple regression with up to 5 predictors, detecting small-to-medium effects (*f*^2^ = 0.15) at α = 0.05 with 80% power required a minimum of 92 participants. For PLS-SEM, following Hair et al. [58], the minimum sample was determined by 10 times the maximum number of structural paths directed at a particular construct (10×3=30 for PCE). Both criteria were comfortably exceeded by the final sample of N = 320.

Participants were randomly assigned to one of eight experimental conditions using Credamo’s built-in randomization function, which employs a uniform distribution algorithm. Randomization was performed automatically upon survey entry, with no researcher intervention. The final distribution was balanced (n per condition ranged from 38 to 41).

#### Procedure.

Participants first read a brief scenario description and were then randomly assigned to view one of the eight product card covers. They were instructed to make judgments as if browsing an e-commerce home feed. After viewing the stimulus, participants completed measures of perceived naturalness, credibility, taste inference, and cognitive engagement. The survey integrated the aforementioned IMCs and demographic verification items to monitor cognitive effort. Finally, participants provided demographic information and received monetary compensation via Credamo. The study took approximately 3–5 minutes to complete.

### Stimuli

The experimental stimuli were designed around the natural food product “apple”, chosen for its broad consumer base, high familiarity, and low perceived risk, thereby minimizing unnecessary cognitive load.

Platform type was operationalized through simulated interface pages and explicit labeling: The content-oriented platform was represented by Xiaohongshu; the transaction-oriented platform by Pinduoduo. These platforms are widely used in natural food consumption contexts and are clearly differentiated as “community-based content browsing” versus “transaction-focused purchasing”.

Image materials were sourced from real platforms and are categorized into two types: (a) natural scenes, depicting apples on branches within orchard settings; (b) lifestyle scenes, showing halved and whole apples arranged on a clean tabletop. All images feature the same apple variety, Red Fuji, to control for product-specific confounding factors.

Text framings were designed in Chinese as follows: (a) production-oriented framing emphasized origin and production process (translated: “Direct from the Orchard! Hand-Picked, Naturally Ripened”); (b) consumption-oriented framing emphasized sensory experience and user appeal (translated: “Crisp, Sweet, and Juicy! Even Picky Eaters Will Love It”). On the transaction-oriented platform (Pinduoduo), textual framings were prefixed with bracketed category labels to match the platform’s typical information architecture (e.g., [Direct from the Orchard]); on the content-oriented platform (Xiaohongshu), these brackets were omitted to match the platform’s more conversational style.

The eight experimental conditions resulted from crossing platform type, image scene, and textual framing: (1) Xiaohongshu / natural scene / production-oriented text; (2) Xiaohongshu / natural scene / consumption-oriented text; (3) Xiaohongshu / lifestyle scene / production-oriented text; (4) Xiaohongshu / lifestyle scene / consumption-oriented text; (5) Pinduoduo / natural scene / production-oriented text; (6) Pinduoduo / natural scene / consumption-oriented text; (7) Pinduoduo / lifestyle scene / production-oriented text; (8) Pinduoduo / lifestyle scene / consumption-oriented text. All brand logos and extraneous visual elements were blurred to ensure that differences among stimuli resulted solely from the manipulated variables.

### Measures

All measures used 7-point Likert scales (1 = strongly disagree, 7 = strongly agree). Items were presented in randomized order across multiple pages to reduce common method bias [59]. Perceived naturalness was assessed with four items adapted from He et al. [60] and Szocs et al. [41], capturing natural atmosphere, green cultivation, ecological environment, and closeness to nature. Perceived credibility was measured with four items from Flanagin and Metzger [11] and Appelman and Sundar [61], assessing information credibility, accuracy, authenticity, and trustworthiness. Taste inference was assessed with five items from Garaus et al. [19] and Liu et al. [62], capturing anticipated taste, flavor, freshness, and appetite stimulation. Product-related cognitive engagement was measured with three items adapted from Jin et al. [63], assessing information-seeking interest and content exploration willingness. Table 1 summarizes all measurement items and their sources.

**Table 1:** Measurement items of latent variables.

Latent variables	Items	Measurements	Adapted from
Perceived Naturalness (PN)	PN1	This cover gives me a sense of natural atmosphere.	[41,60]
	PN2	This cover reminds me of green cultivation or natural farming.	
	PN3	The environment shown on the cover looks ecological.	
	PN4	The image and title on the cover make me feel close to nature.	
Perceived Credibility (PC)	PC1	I think the product information shown on the cover is credible.	[11,61]
	PC2	I believe the product features mentioned on the cover are accurate.	
	PC3	The overall cover conveys a sense of authenticity and reliability.	
	PC4	This cover makes me feel that it can be trusted.	
Taste Inference (TI)	TI1	This product looks delicious and appetizing.	[19,62]
	TI2	The cover makes me think this product would taste good.	
	TI3	I imagine the product tastes fresh and flavorful.	
	TI4	After viewing the cover, I look forward to how this product might taste.	
	TI5	The product shown and described on the cover stimulates my appetite and makes me want to taste it.	
Product-related Cognitive Engagement (PCE)	PCE1	I would like to learn more detailed information about this product.	[63]
	PCE2	I have developed a strong interest in this product.	
	PCE3	I am highly willing to explore more content about this product.	

### Analytical strategy

To address the inherent limitations of any single analytical approach, this study adopts methodological triangulation integrating partial least squares structural equation modeling (PLS-SEM), multi-group analysis (MGA), and necessary condition analysis (NCA) [58,64,65]. This combination is theoretically synergistic: PLS-SEM tests sufficiency-based path relationships; MGA examines the boundary conditions of these relationships across experimentally manipulated contexts; and NCA identifies the minimum necessary levels of predictors required to achieve high outcome states. Together, these methods map the sufficient, contextually bounded, and necessary dimensions of the cognitive processing chain, respectively—a tripartite analytical architecture that mirrors the theoretical distinction between causal sufficiency, moderation, and necessity in the philosophy of science [65]. Table 2 summarizes the distinctive contributions of each method and their interpretive logics.

**Table 2:** Integration of analytical perspectives: A diagnostic framework.

Analytical Dimension	Method	Core Question	Interpretive Logic
Sufficiency	PLS-SEM	Does X contribute to Y when other factors are controlled?	β and *R*^2^ quantify average linear contribution; bootstrapping tests statistical significance
Boundary	MGA	When (under what conditions) does X → Y hold or vary?	Path coefficient differences across experimental groups reveal moderating contexts; MICOM ensures measurement invariance
Necessity	NCA	Must X reach a minimum level for high Y to be possible?	Ceiling line identifies the minimum required level of X for each percentile of Y; effect sizes distinguish bottleneck variables from facilitators

Note: This framework distinguishes between what drives outcomes on average (sufficiency), when these drivers matter (boundary), and what must be present for high performance (necessity), respectively. Convergence across methods strengthens causal inference; divergence indicates that a predictor operates differently across analytical lenses (e.g., a variable may be sufficient but not necessary, or necessary only in certain contexts).

#### PLS-SEM.

Conducted using SmartPLS to assess measurement model reliability and validity and estimate latent variable path relationships. PLS-SEM was selected over covariance-based SEM (CB-SEM) for three reasons specific to this research context. First, the study prioritizes predictive accuracy and path coefficient estimation over strict parameter recovery, as the theoretical goal is to forecast cognitive engagement from interface cues rather than to confirm a pre-specified population covariance structure [58]. Second, the model includes a formative-like experimental antecedent (perceived naturalness absorbing three manipulated factors) that violates the reflective measurement assumptions underlying CB-SEM’s maximum likelihood estimation. Third, the sample size (N = 320), while adequate for PLS-SEM’s partial least squares algorithm, approaches the lower boundary for stable CB-SEM solutions given the model’s complexity (14 indicators, 4 latent constructs, and 8 control paths). These considerations render PLS-SEM not merely a convenient alternative but the analytically appropriate choice for the study’s explanatory objectives.

All constructs in the structural model are reflectively measured: indicators are conceptualized as manifestations of underlying latent constructs rather than their causal antecedents [66]. Accordingly, Mode A (correlation-based indicator weights) was employed as the standard estimation approach for reflective measurement models, as it maximizes the correlation between the composite and its indicators and prioritizes measurement reliability [67]. Mode B (regression-based weights), designed for formative measurement models where indicators cause the construct, was not theoretically appropriate for this study’s measurement specifications. A sensitivity analysis comparing Mode A and Mode B estimation is reported in Supporting Information (S1 File) to address potential concerns about estimation mode choice.

In the PLS-SEM analysis, the three manipulated factors are not entered as exogenous variables in the structural model. Instead, their variance is absorbed into the measured construct of perceived naturalness, which serves as the entry point of the experimental effects into the cognitive processing chain. This approach is consistent with the theoretical framework: the manipulations represent surface-level interface features, whereas perceived naturalness represents the underlying psychological mechanism that transmits these features to downstream cognition. The structural model therefore estimates the relationships among the four latent constructs, while the experimental conditions’ moderating roles are examined through multi-group analysis.

#### Multi-group analysis (MGA).

Performed in SmartPLS to test whether path coefficients differed significantly across experimentally manipulated groups (platform type, cover image scene, and textual framing). MGA was preferred over product-term moderation analysis for two substantive reasons. First, the experimental manipulations constitute categorical group-defining variables with no natural ordering, making product-term interactions statistically inefficient and substantively uninterpretable. Second, MGA allows for the simultaneous testing of measurement invariance (via MICOM) and structural path differences, whereas product-term approaches assume measurement equivalence across moderator levels. By estimating separate models per group and comparing path coefficients directly, MGA provides a conservative and transparent test of the moderating effects.

#### Necessary condition analysis (NCA).

Conducted in R using the NCA package, NCA was selected over qualitative comparative analysis (QCA) because the research objective is to identify whether individual antecedents function as necessary conditions at different stages of the proposed cognitive process, rather than to uncover conjunctural sufficient configurations. While QCA is well suited for examining asymmetric combinations of conditions, it does not assess the independent necessity of single factors nor quantify their constraining effects.

In line with the theorized hierarchical cognitive chain, NCA was applied not only to the final outcome (product-related cognitive engagement) but also to intermediate constructs (e.g., taste inference) to examine whether each stage constitutes a potential bottleneck. This stepwise application allows for testing whether minimum levels of lower-stage perceptual judgments are required for higher-stage cognitive responses to emerge.

Following established procedures [65], effect sizes were estimated using both Ceiling Envelopment (CE-FDH) and Ceiling Regression (CR-FDH) techniques. Statistical significance was assessed via a permutation test with 10,000 resamples [64,68]. In addition, bottleneck tables were computed to identify the minimum required levels of each condition at different outcome percentiles, enabling a more fine-grained interpretation of constraint effects across the distribution.

## Results

### Descriptive statistics and measurement model

#### Sample characteristics.

Table 3 presents the demographic characteristics of the final sample. The majority of participants were aged 26–35 (46.6%), followed by 18–25 (34.1%), 36–45 (13.4%), 46–55 (4.4%), and over 55 (1.6%). Female participants accounted for 60.9%, while males accounted for 39.1%. Regarding education, 68.8% held a bachelor’s degree, 14.4% had a master’s degree, 12.2% had an associate degree, and 4.7% had a high school diploma or lower. In terms of annual disposable income, 15.9% reported earning 10,000–50,000 RMB, 78.1% earned 60,000–120,000 RMB, and 6.0% earned more than 120,000 RMB. For platform usage frequency, 31.6% used the platform multiple times daily, 31.3% used it 3–5 times per week, 18.8% used it once daily, 17.8% used it 1–2 times per week, and 0.6% rarely used it. Regarding the frequency of agricultural product purchases, 38.8% purchased occasionally, 35.9% purchased frequently, 16.3% purchased infrequently, 5.9% purchased very frequently, and 3.1% never purchased.

**Table 3:** Sample demographic characteristics.

Variable and category	Frequency	Percentage (%)	Variable and category	Frequency	Percentage (%)
**Gender**			**Annual disposable income (RMB / Year)**		
Female	195	60.9	10,000–50,000	51	15.9
Male	125	39.1	60,000–120,000	250	78.1
			Above 120,000	19	6.0
**Age (Years)**			**Education level**		
18–25	109	34.1	High school / Vocational / Technical	15	4.7
26–35	149	46.6	Associate degree	39	12.2
36–45	43	13.4	Bachelor’s degree	220	68.7
46–55	14	4.4	Master’s degree	46	14.4
Over 55	5	1.5			
**Platform browsing or usage frequency**			**Frequency of purchasing on the platform**		
Rarely or never	2	0.6	Never	10	3.1
1–2 times per week	57	17.8	Occasionally	52	16.3
3–5 times per week	100	31.2	Sometimes	124	38.8
About once per day	60	18.8	Frequently	115	35.9
Multiple times per day	101	31.6	Very frequently	19	5.9

#### Manipulation checks.

To verify that the manipulated stimuli effectively conveyed the intended perceptual cues, manipulation checks were conducted on the three independent variables prior to the main measurements.

For platform type, participants answered the question, “Which type of platform do you think Pinduoduo/Xiaohongshu leans toward? (1 = strongly transaction-oriented, 7 = strongly content-oriented)” after reading the platform descriptions. Results showed that the Xiaohongshu group (M = 5.43) scored significantly higher than the Pinduoduo group (M = 3.26, t(288.297) = 11.308, p < 0.001), and there was no significant difference between groups in familiarity (p = 0.428).

For the image scenario manipulation check, participants responded to the question, “Which style do you think this image leans toward? (1 = strongly lifestyle-oriented, 7 = strongly nature-oriented)”. Results confirmed a significant difference between the lifestyle group (M = 2.93) and the natural group (M = 5.59, t(296.12) = 15.225, p < 0.001), and there was no significant difference in image preference between groups (p = 0.508), ruling out the influence of preference bias.

For the textual framing, participants answered, “Which aspect does the title emphasize more? (1 = strongly emphasizes production process, 7 = strongly emphasizes consumption experience)”. Results also indicated successful manipulation ($M_{production}$ = 2.86, $M_{consumption}$ = 5.94, t(273.877) = −17.250, p < 0.001).

These results confirm that the experimental manipulations effectively conveyed the intended perceptual cues. With the validity of the manipulations established, the analysis shifts from experimentally manipulated factors to the assessment of latent psychological constructs.

#### Measurement model assessment.

Following the confirmation of manipulation effectiveness, the measurement model was first assessed to ensure the reliability and validity of the key latent constructs—perceived naturalness, perceived credibility, taste inference, and product-related cognitive engagement—before proceeding to the structural model analysis. To evaluate the reliability of the measurement model, PLS-SEM analysis was conducted using SmartPLS. Results in Table 4 indicate that Cronbach’s alpha (α) and composite reliability (CR) for all latent constructs exceed 0.7, and the average variance extracted (AVE) for each construct is above 0.5, meeting the recommended thresholds. These results suggest good internal consistency and convergent validity of the scales. All indicator loadings are above 0.708, and variance inflation factors (VIFs) are below 3.3, indicating no significant issues with multicollinearity. Results in Table 5 show that discriminant validity meets the Fornell-Larcker criterion (the square root of AVE is greater than the inter-construct correlations) and the HTMT ratio (all values are below 0.90), further supporting the validity of the measurement model.

**Table 4:** Structural reliability and validity results.

Latent constructs and indicators	Mean	SD	Loadings (>0.708)	VIF (<3.3)	α (>0.7)	CR (>0.7)	AVE (>0.5)
Perceived Naturalness (PN)					0.891	0.924	0.753
PN1	5.775	1.143	0.845	2.176			
PN2	5.578	1.237	0.873	2.307			
PN3	5.656	1.323	0.868	2.470			
PN4	5.594	1.256	0.885	2.563			
Perceived Credibility (PC)					0.820	0.881	0.650
PC1	5.456	0.921	0.810	1.732			
PC2	5.622	0.999	0.769	1.589			
PC3	5.616	0.952	0.808	1.705			
PC4	5.528	0.935	0.837	1.905			
Taste Inference (TI)					0.830	0.880	0.595
TI1	5.925	0.866	0.773	1.703			
TI2	5.978	0.979	0.742	1.547			
TI3	5.809	0.954	0.729	1.562			
TI4	5.794	0.978	0.791	1.697			
TI5	5.831	1.026	0.819	1.891			
Product-related Cognitive Engagement (PCE)					0.824	0.895	0.740
PCE1	5.850	0.950	0.826	1.739			
PCE2	5.709	1.087	0.857	1.858			
PCE3	5.619	1.150	0.897	2.292			

VIF = Variance inflation factor statistics, α = Cronbach’s alpha, CR = Composite reliability (rho_c), AVE = Average variance extracted.

**Table 5:** Discriminant validity results.

Fornell-Larcker criterion / HTMT	PN	PC	PCE	TI
Perceived Naturalness (PN)	0.868	0.537	0.563	0.547
Perceived Credibility (PC)	0.625	0.806	0.853	0.821
Product-related Cognitive Engagement (PCE)	0.485	0.701	0.860	0.889
Taste Inference (TI)	0.475	0.680	0.740	0.772

Values on the diagonal represent the square root of the AVE; values below the diagonal are the correlations between latent variables; values above the diagonal are the HTMT (Heterotrait-Monotrait ratio of correlations) ratios.

Beyond these statistical indicators, we conducted additional procedural and statistical assessments to address potential common method bias (CMB), given that all measures were self-reported. Procedurally, randomized item order and multi-page questionnaire design were employed to reduce respondents’ ability to infer relationships among constructs and to mitigate consistency motifs. Statistically, multiple complementary tests were conducted. First, Harman’s single-factor test showed that the first unrotated factor accounted for 47.2% of the total variance, below the conventional 50% threshold. Second, following the full collinearity assessment approach in PLS-SEM, all variance inflation factors (VIFs) were below the conservative threshold of 3.3, indicating that CMB is unlikely to be a serious concern. Importantly, the observed pattern of relationships is inconsistent with a generalized positivity or acquiescence bias explanation. If such bias were driving the results, one would expect uniformly high correlations across constructs. However, the empirical results exhibit differentiated effect sizes and explanatory power across endogenous variables, suggesting that the observed relationships reflect substantive structural associations rather than a common response tendency. Having established satisfactory reliability, validity, and method bias controls for the measurement model, the next step is to examine the structural relationships among the latent constructs and test the proposed hypotheses.

The discriminant validity results in Table 5 align with the theoretical distinctions articulated. The correlation between perceived credibility and taste inference (r = 0.680) is moderate rather than near-perfect, consistent with the conceptualization that credibility concerns truth verification whereas taste inference involves experiential simulation—qualitatively different cognitive operations. Similarly, the correlation between taste inference and cognitive engagement (r = 0.740), while stronger, remains below the 0.85 threshold for construct redundancy [69], supporting the proposition that anticipated sensory pleasure (taste) and motivational readiness to act (engagement) are distinct stages in the cognitive-affective-conative sequence. The weakest correlation between perceived naturalness and cognitive engagement (r = 0.485) further validates the hierarchical model, wherein the initial perceptual trigger is relatively distal from the final motivational outcome.

To provide additional empirical evidence for construct distinctiveness, we examined cross-loadings of indicators on non-assigned constructs. All indicator loadings on their theoretically assigned constructs exceed 0.70, while loadings on non-assigned constructs are substantially lower (all cross-loadings < 0.67). The highest cross-loading occurs for PCE items on TI (0.530–0.648) and TI items on PCE (0.530–0.648), which is expected given the motivational proximity of taste anticipation to engagement intention; nonetheless, these values remain markedly below the loadings on the assigned constructs (0.729–0.898 for PCE, 0.729–0.819 for TI). This pattern supports the empirical uniqueness of each construct and mitigates concerns that the constructs merely represent adjacent stages of an undifferentiated evaluative process.

### Structural model and hypothesis testing

The structural model was evaluated using the PLS-SEM algorithm and bootstrapping to assess model fit and the significance of path coefficients. Model fit results indicate that the standardized root mean square residual (SRMR) is 0.057, below the 0.08 threshold, suggesting a good fit. The unweighted least squares discrepancy (d_ULS) and the geodesic distance (d_G) also support the model’s adequacy. Regarding predictive capability, product-related cognitive engagement shows the highest explanatory power (*R*^2^ = 0.643), while taste inference and perceived credibility have *R*^2^ values of 0.479 and 0.289, respectively. In addition, the predictive relevance of the model was assessed using Stone-Geisser’s *Q*^2^ via blindfolding [70]. All endogenous constructs exhibited *Q*^2^ values greater than zero (PC: *Q*^2^ = 0.184; TI: *Q*^2^ = 0.281; PCE: *Q*^2^ = 0.468), indicating acceptable out-of-sample predictive capability. Overall, these results indicate that the model has strong predictive validity.

#### Direct and indirect effects.

**Fig 2:**
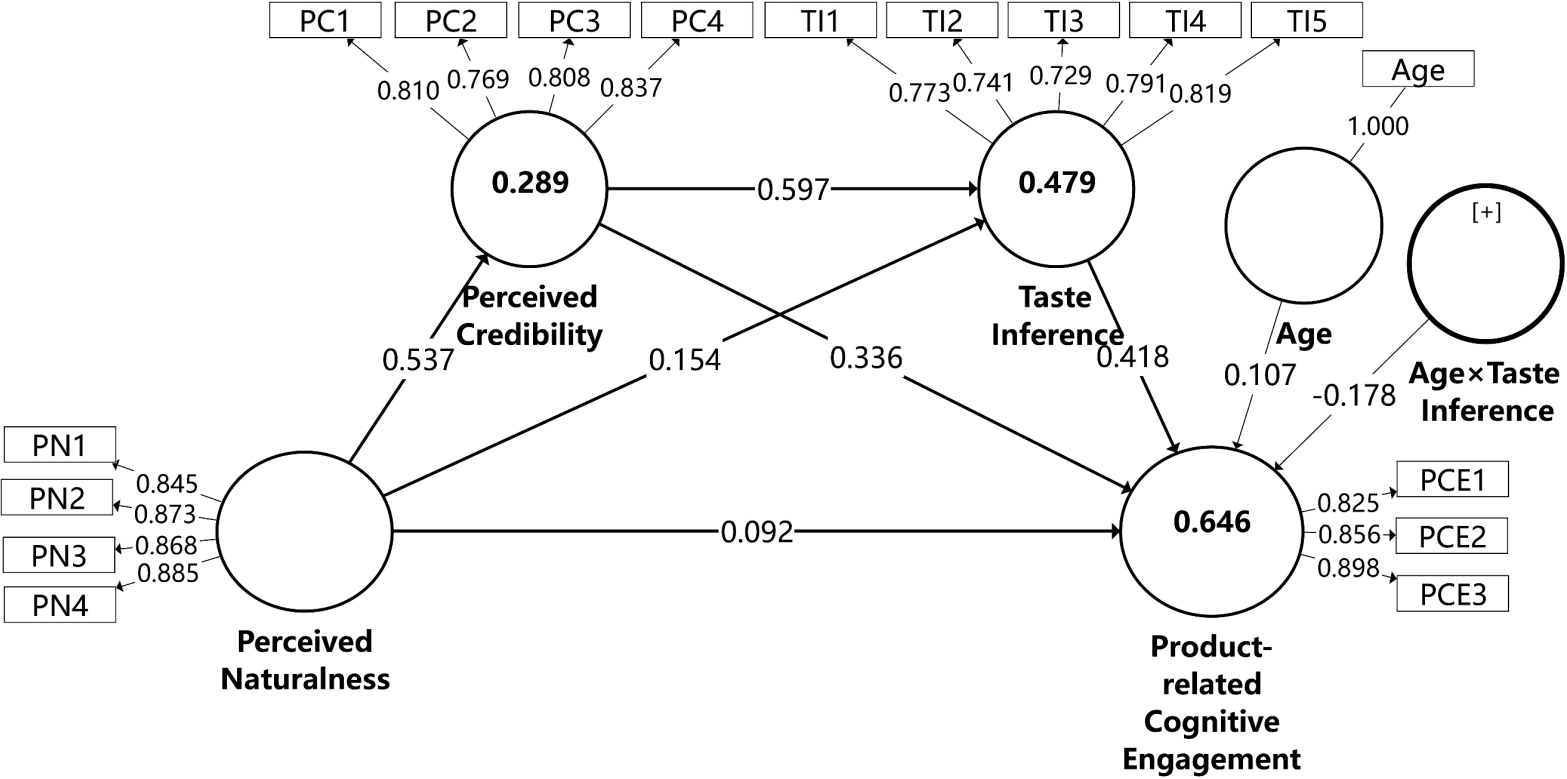
The structural model with path coefficients, loadings, and *R*^2^ values.

Path coefficient analysis results (see Fig 2 and Table 6) indicate that the proposed research model is empirically supported. Specifically, perceived naturalness was found to exert a significant direct positive effect on product-related cognitive engagement (β = 0.092, 95% CI [0.009, 0.183], p = 0.039, *f*^2^ = 0.016, small effect), providing empirical evidence for H 1. The analysis also reveals that taste inference (β = 0.422, 95% CI [0.313, 0.543], p < 0.001, *f*^2^ = 0.238, medium-to-large effect) and credibility (β = 0.336, 95% CI [0.200, 0.459], p < 0.001, *f*^2^ = 0.152, medium effect) have strong positive effects on product-related cognitive engagement. Perceived naturalness significantly strengthens credibility (β = 0.537, 95% CI [0.425, 0.637], p < 0.001, *f*^2^ = 0.406, large effect) and taste inference (β = 0.154, 95% CI [0.056, 0.257], p = 0.002, *f*^2^ = 0.033, small effect). Furthermore, the effect of credibility on taste inference is the most pronounced (β = 0.597, 95% CI [0.495, 0.685], p < 0.001, *f*^2^ = 0.487, large effect).

**Table 6:** Path coefficient results.

Direct Effects	β	t-value	p-value	CI [2.5%−97.5%]		$f^{2}$
PN → PC	0.537	9.826^***^	0.000	0.425	0.637	0.406
PN → PCE	0.092	2.069^*^	0.039	0.009	0.183	0.016
PN → TI	0.154	3.030^**^	0.002	0.056	0.257	0.033
PC → PCE	0.336	5.037^***^	0.000	0.200	0.459	0.152
PC → TI	0.597	12.350^***^	0.000	0.495	0.685	0.487
TI → PCE	0.422	7.074^***^	0.000	0.313	0.543	0.238
Age → PCE	0.104	2.299^*^	0.022	0.012	0.191	0.027
Age × TI → PCE	−0.142	2.807^**^	0.005	−0.233	−0.036	0.049

PN = Perceived Naturalness; PC = Perceived Credibility; PCE = Product-related Cognitive Engagement; TI = Taste Inference. ^***^ indicates p < 0.001; ^**^ indicates 0.001 ≤ p < 0.01; ^*^ indicates 0.01 ≤ p < 0.05. *f*^2^ effect sizes: ≥ 0.35 = large, ≥ 0.15 = medium, ≥ 0.02 = small [71].

The mediation results presented in Table 7 further support the proposed mechanisms. Perceived naturalness has a significant indirect effect on cognitive engagement through two parallel single mediation paths: via taste inference (β = 0.065, p = 0.005, VAF = 13.74%) and via credibility (β = 0.181, p < 0.001, VAF = 38.27%), supporting H 2 and 3. Additionally, the sequential mediation path (PN → PC → TI → PCE) is significant (β = 0.135, p < 0.001, VAF = 28.54%), supporting H 4. The differential VAF values across these paths provide evidence for construct distinctiveness. If credibility, taste inference, and engagement were merely part of an undifferentiated process, the indirect effects would be equivalent. However, the dominance of the credibility path (VAF = 38.27%) over the taste inference path (VAF = 13.74%) indicates that they contribute unique explanatory variance, consistent with their distinct cognitive functions: truth verification versus experiential simulation. The substantial sequential mediation (VAF = 28.54%) demonstrates that these constructs operate as complementary mechanisms, where credibility serves as an attributional gatekeeper for subsequent sensory imagination. Finally, perceived naturalness has a strong indirect effect on taste inference via credibility (β = 0.321, p < 0.001, VAF = 67.86%), and credibility itself exerts a significant indirect effect on engagement through taste inference (β = 0.252, p < 0.001, VAF = 42.86%).

**Table 7:** Mediation effects (bootstrapping) results.

Specific indirect effect path	β	t-value	p-value	CI [2.5%−97.5%]		Significance	VAF
PN → PC → PCE	0.181	4.256	0.000	0.101	0.267	^***^	38.27%
PN → TI → PCE	0.065	2.783	0.005	0.024	0.115	^**^	13.74%
PN → PC → TI → PCE	0.135	5.038	0.000	0.089	0.194	^***^	28.54%
PN → PC → TI	0.321	7.718	0.000	0.243	0.403	^***^	67.86%
PC → TI → PCE	0.252	5.562	0.000	0.171	0.347	^***^	42.86%

PN = Perceived Naturalness; PC = Perceived Credibility; TI = Taste Inference; PCE = Product-related Cognitive Engagement. ^***^ indicates p < 0.001; ^**^ indicates 0.001 ≤ p < 0.01; ^*^ indicates 0.01 ≤ p < 0.05. VAF (Variance Accounted For) measures the proportion of variance in the dependent variable explained by the mediation (indirect) effects. VAF is calculated as the ratio of the indirect effect to the total effect (VAF = Indirect Effect / Total Effect).

To verify that the sequential mediation structure is not an artifact of model specification, we tested an alternative model in which the sequential path between the two mediators was removed (i.e., no path from perceived credibility to taste inference), thereby specifying perceived credibility and taste inference as simultaneous rather than sequential mediators. In this alternative model, perceived naturalness exerts direct effects on both mediators and the outcome (i.e., PC, TI, and PCE), while the two mediators each independently predict product-related cognitive engagement without the proposed causal ordering. This alternative specification yielded a poorer model fit (SRMR = 0.125, exceeding the recommended threshold of 0.08) and substantially lower explanatory power for taste inference (*R*^2^ = 0.227 vs. 0.479 in the proposed sequential model). The significant deterioration in both model fit and explanatory power provides strong support for the theoretical appropriateness of the sequential mediation structure over the direct-effects alternative.

#### Moderating effects of experimental factors.

While the overall structural model treats the experimental manipulations as antecedent conditions channeled through perceived naturalness, the multi-group analysis (MGA) examines whether the strength of the structural pathways among the latent constructs varies across these experimental conditions. This analysis addresses whether platform type, image scene, and textual framing act as boundary conditions that moderate the structural relationships among the latent constructs.

In accordance with the procedural requirements of multi-group analysis (MGA), this study systematically assessed the measurement invariance of composite models (MICOM) before conducting cross-group comparisons. The results of the three-stage invariance test are presented in  Tables 8–10. Full or partial measurement invariance was successfully established across all grouping dimensions (platform type, image scene, and textual framing), thereby ensuring that observed structural differences stem from substantive moderating effects rather than measurement bias. Following established recommendations for partial least squares analysis [72], these results provide the necessary statistical foundation and theoretical support for subsequent cross-group comparisons.

**Table 8:** MICOM assessment for platform type (Content-oriented – Transaction-oriented).

	Step 1	Step 2			Step 3 (a)				Step 3 (b)			
Variable	Configural invariance	OC	5.0%	Perm p-value	MOD	2.5%	97.5%	Perm p-value	VOD	2.5%	97.5%	Perm p-value
Age	Yes	1.000	1.000	0.052	−0.136	−0.207	0.217	0.233	−0.117	−0.426	0.394	0.578
Age × TI	Yes	0.971	0.893	0.498	0.041	−0.187	0.166	0.666	0.234	−0.688	0.654	0.517
PC	Yes	1.000	0.999	0.480	−0.090	−0.221	0.217	0.430	0.121	−0.410	0.410	0.578
PN	Yes	1.000	0.998	0.677	0.024	−0.234	0.213	0.848	0.081	−0.452	0.451	0.717
PCE	Yes	0.999	0.999	0.068	−0.102	−0.225	0.213	0.399	0.744	−0.532	0.549	0.006
TI	Yes	0.997	0.997	0.041	−0.181	−0.223	0.227	0.117	0.522	−0.527	0.531	0.056

OC is original correlation, Perm is permutation, MOD is mean – original difference, VOD is variance – original difference.

**Table 9:** MICOM assessment for cover image scene (Lifestyle – Natural).

	Step 1	Step 2			Step 3 (a)				Step 3 (b)			
Variable	Configural invariance	OC	5.0%	Perm p-value	MOD	2.5%	97.5%	Perm p-value	VOD	2.5%	97.5%	Perm p-value
Age	Yes	1.000	1.000	0.177	0.092	−0.219	0.233	0.375	0.087	−0.397	0.401	0.665
Age × TI	Yes	0.981	0.882	0.691	−0.061	−0.176	0.169	0.487	−0.414	−0.632	0.650	0.218
PC	Yes	1.000	0.999	0.504	0.303	−0.218	0.230	0.006	−0.267	−0.411	0.438	0.207
PN	Yes	1.000	0.998	0.633	−0.426	−0.216	0.215	/	0.642	−0.455	0.434	0.005
PCE	Yes	1.000	0.999	0.772	0.305	−0.214	0.224	0.006	−0.796	−0.532	0.501	0.003
TI	Yes	0.999	0.997	0.399	0.333	−0.227	0.235	0.002	−0.441	−0.495	0.506	0.089

OC is original correlation, Perm is permutation, MOD is mean – original difference, VOD is variance – original difference.

**Table 10:** MICOM assessment for textual communication framing (Consumption-oriented – Production-oriented).

	Step 1	Step 2			Step 3 (a)				Step 3 (b)			
Variable	Configural invariance	OC	5.0%	Perm p-value	MOD	2.5%	97.5%	Perm p-value	VOD	2.5%	97.5%	Perm p-value
Age	Yes	1.000	1.000	/	−0.273	−0.217	0.207	0.014	−0.062	−0.394	0.416	0.759
Age × TI	Yes	0.942	0.884	0.211	0.048	−0.175	0.178	0.576	0.264	−0.697	0.676	0.472
PC	Yes	1.000	0.999	0.411	−0.277	−0.255	0.209	0.020	0.167	−0.399	0.437	0.457
PN	Yes	0.999	0.998	0.170	−0.181	−0.223	0.223	0.112	0.148	−0.472	0.428	0.542
PCE	Yes	1.000	0.999	0.920	−0.291	−0.235	0.224	0.019	0.370	−0.521	0.538	0.178
TI	Yes	1.000	0.997	0.861	−0.365	−0.240	0.229	/	0.301	−0.521	0.504	0.281

OC is original correlation, Perm is permutation, MOD is mean – original difference, VOD is variance – original difference.

**Fig 3:**
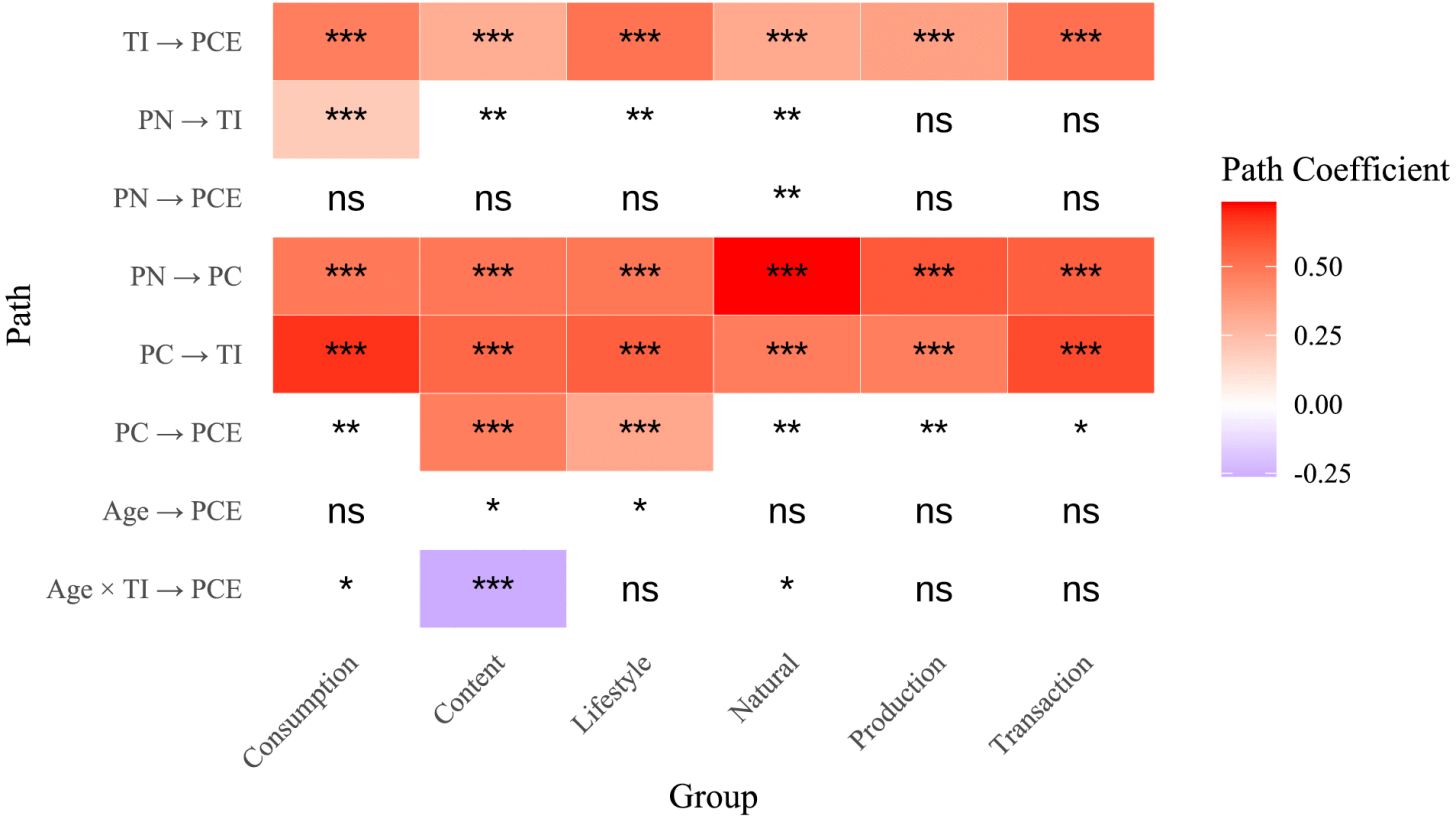
Heatmap of path coefficients and significance differences across conditions. Note: PN = Perceived Naturalness; PC = Perceived Credibility; TI = Taste Inference; PCE = Product-related Cognitive Engagement. ^***^ indicates p < 0.001; ^**^ indicates 0.001 ≤ p < 0.01; ^*^ indicates 0.01 ≤ p < 0.05; ${\;}^{ns}$ indicates p ≤ 0.05. The color gradient represents the magnitude and direction of the path coefficients: purple indicates negative coefficients, with darker shades representing larger negative values; white indicates coefficients close to zero; red indicates positive coefficients, with darker shades representing larger positive values.

The MGA results (Table 11) and the corresponding heatmap (Fig 3) indicate that platform type, cover image scene, and textual framing significantly moderate the structural paths within the model.

**Table 11:** MGA results (paths with significant differences in path coefficients).

Variable and path	Path coefficient difference	Two-tailed p-value	β		p-value		Results
Platform Type	(1) – (2)	(1) vs. (2)	(1)	(2)	(1)	(2)	
PC → PCE	−0.261^*^	0.044	0.214^*^	0.475^***^	0.016	0.000	All significant, stronger on the content-oriented platform.
Age × TI → PCE	0.196^*^	0.038	−0.065 ${\;}^{ns}$	−0.261^***^	0.372	0.000	Significant only on the content-oriented platform.
**Image Scene**	**(3) – (4)**	**(3) vs. (4)**	**(3)**	**(4)**	**(3)**	**(4)**	
PN → PC	−0.234^*^	0.013	0.499^***^	0.733^***^	0.000	0.000	All significant, stronger for natural scene images.
PN → PCE	−0.259^*^	0.015	0.026 ${\;}^{ns}$	0.285^**^	0.636	0.002	Significant only for natural scene images.
**Textual Framing**	**(5) – (6)**	**(5) vs. (6)**	**(5)**	**(6)**	**(5)**	**(6)**	
PC → TI	0.207^*^	0.024	0.681^***^	0.474^***^	0.000	0.000	All significant, stronger under the consumption-oriented framing.

(1) = Transaction; (2) = Content; (3) = Lifestyle; (4) = Natural; (5) = Consumption; (6) = Production; PN = Perceived Naturalness; PC = Perceived Credibility; TI = Taste Inference; PCE = Product-related Cognitive Engagement. ^***^ indicates p < 0.001; ^**^ indicates 0.001 ≤ p < 0.01; ^*^ indicates 0.01 ≤ p < 0.05; ${\;}^{ns}$ indicates p ≤ 0.05.

Platform type as a boundary condition. On content-oriented platforms, the effect of perceived credibility on product-related cognitive engagement is significantly stronger (β = 0.475, p < 0.001) than on transaction-oriented platforms (β = 0.214, p < 0.05), with a significant path difference (Δβ=−0.261, p = 0.044). This divergence suggests that content-centric environments (e.g., Xiaohongshu) function as high-involvement contexts where credibility is highly diagnostic of engagement intentions. Conversely, on transaction-heavy platforms like Pinduoduo, price and efficiency cues may overshadow the impact of credibility. This pattern aligns with the ELM, supporting H 5.

Image scene as a contextual enhancer. The moderation analysis for image scenes reveals that natural backgrounds significantly amplify the persuasive power of naturalness cues. Specifically, the path from perceived naturalness to perceived credibility (PN → PC) is markedly stronger in the natural scene group (β = 0.733, p < 0.001) than in the lifestyle group (β = 0.499, p < 0.001), with a significant difference (Δβ=−0.234, p = 0.013). Notably, the direct effect of perceived naturalness on cognitive engagement (PN → PCE) is only significant under natural scene conditions (β = 0.285, p = 0.002), while remaining non-significant for lifestyle images. These results indicate that environmental congruency validates product attributes, thereby supporting H 6.

Textual framing and cognitive links. Finally, textual framing demonstrates a significant moderating effect on the link between credibility and sensory inference. The relationship between perceived credibility and taste inference (PC → TI) is more pronounced under consumption-oriented framing (β = 0.681, p < 0.001) than under production-oriented framing (β = 0.474, p < 0.001), with a significant difference (Δβ = 0.207, p = 0.024). This suggests that experiential language lowers the cognitive hurdle for mental simulation, allowing users to more readily translate established trust into sensory anticipation, providing empirical support for H 7.

These results demonstrate that the experimental manipulations moderate the structural relationships among the latent constructs, confirming their role as boundary conditions in the cognitive processing chain. The experimental design ensures that these boundary effects are attributable to the manipulated interface features rather than to self-selection or confounding variables, strengthening the causal interpretation of the moderated relationships.

### Supplementary analyses

#### Exploratory analysis: Age as a moderator.

In addition to the hypothesized moderating effects of experimental factors, an exploratory analysis examined whether demographic variables act as boundary conditions for the structural paths. Among the demographic factors tested (sex, education level, income, platform usage frequency, and purchase frequency), only age emerged as a significant moderator.

**Fig 4:**
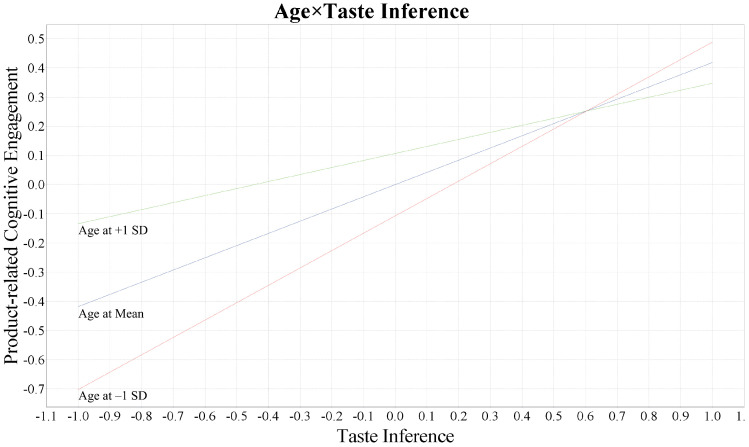
Simple slopes analysis for the moderating effect of age on the relationship between taste inference and product-related cognitive engagement.

Specifically, the results indicate that age exerts a significant negative moderating effect on the path from taste inference to product-related cognitive engagement (β=−0.142, p < 0.01). As illustrated by the simple slopes in Fig 4, the positive influence of taste inference on cognitive engagement diminishes as consumer age increases. Interestingly, this moderating effect of age exhibits significant cross-platform variation (Table 11). The interaction effect of Age × Taste Inference on cognitive engagement is highly significant on content-oriented platforms (β=−0.261, p < 0.001), while remaining non-significant on transaction-oriented platforms (β=−0.065, p = 0.372), with a significant group difference (Δβ = 0.196, p = 0.038).

These findings suggest that the influence of age on the translation of sensory heuristics into engagement is highly context-dependent. On content-centric platforms (e.g., Xiaohongshu) where elaborative processing is normative, older consumers may exhibit lower sensitivity to taste-based appeals, potentially relying more on accumulated product knowledge or analytical skepticism. In contrast, on transaction-driven platforms (e.g., Pinduoduo) where efficiency and goal-attainment are prioritized, consumers across different age groups appear to adopt a more uniform, goal-oriented processing style. This goal-congruent behavior effectively neutralizes the moderating influence of age on the taste-engagement link, as both younger and older cohorts prioritize transaction completion over sensory exploration.

#### NCA.

While MGA focuses on differences in path strengths across groups from a sufficiency perspective, it does not address whether certain conditions are necessary for achieving high levels of the outcome variables. Therefore, NCA is conducted as a supplementary exploratory tool to identify critical bottleneck conditions.

NCA examines necessity logic—whether a minimum level of an antecedent is required for an outcome to occur at all—which complements PLS-SEM’s focus on average effects (sufficiency logic). This distinction is theoretically grounded in hierarchical information processing theories, which posit that lower-level perceptual processing must reach a threshold before higher-level cognitive elaboration can proceed [73]. The “thresholds” identified by NCA are empirically derived ceiling lines based on observed data distributions [65]. They reflect data-driven bottleneck points in the perceptual-cognitive processing chain within this specific sample.

**Fig 5:**
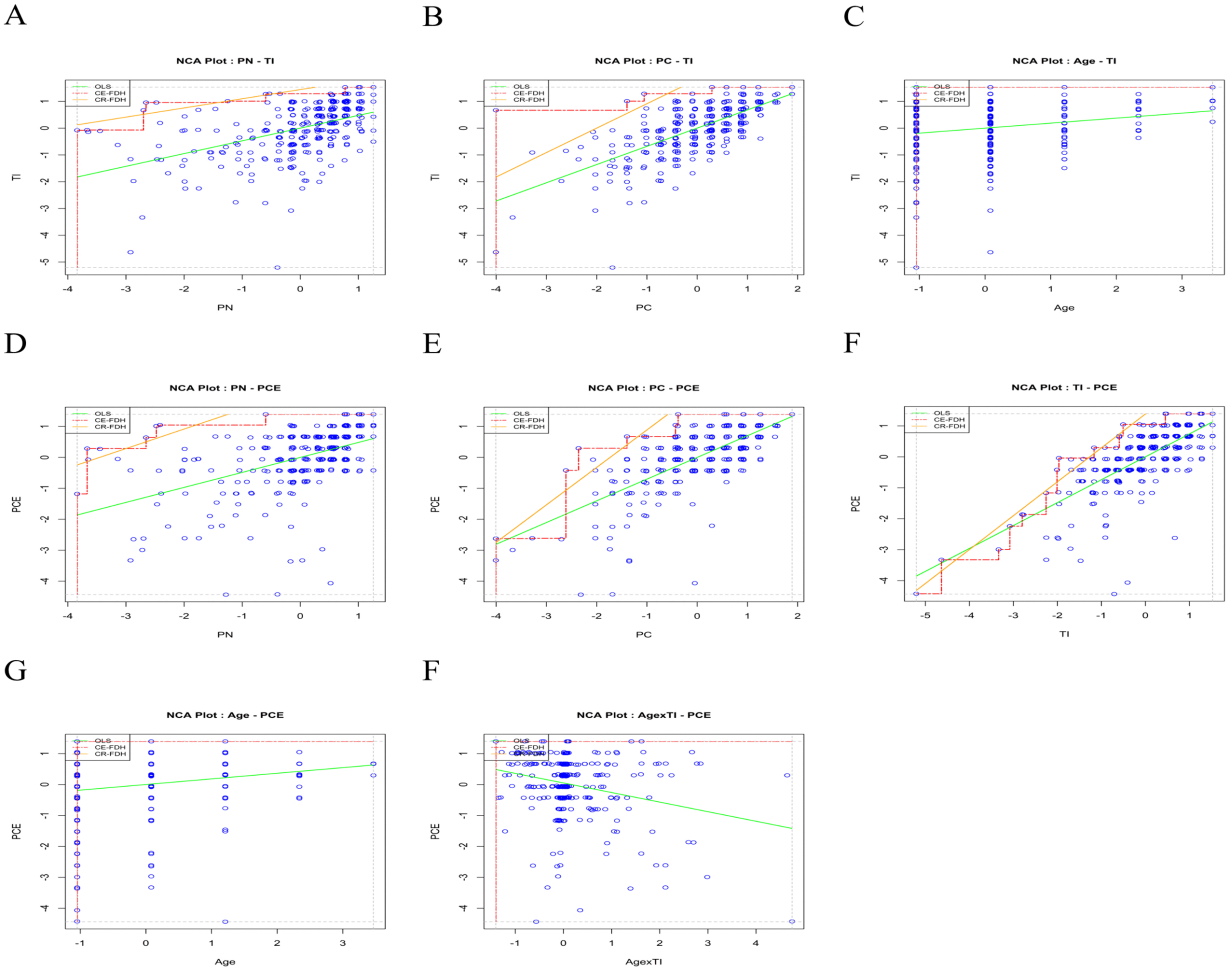
NCA scatter plots. Note: The NCA plots A, B, C in Row 1 illustrate the necessary conditions between perceived naturalness (PN), perceived credibility (PC), age, and taste inference (TI); D, E, F in Row 2 shows how these variables, including TI, relate to product-related cognitive engagement (PCE); G, H in Row 3 highlights the effects of age and the age × TI interaction on PCE.

The NCA results (see Table 12) indicate that taste inference is the most critical necessary condition for product-related cognitive engagement, showing the largest effect size (CE-FDH = 0.414, p < 0.001). Credibility judgment and perceived naturalness were also identified as necessary conditions, though with weaker effects, especially for perceived naturalness (CE-FDH = 0.079, p = 0.014). Specifically, credibility demonstrated stronger necessity under the CE-FDH method (0.156, p < 0.001), while under the CR-FDH method, its effect size ranged from 0.1 ≤ d ≤ 0.3, indicating a moderate effect. The ceiling line analysis further revealed that perceived naturalness and credibility exerted significant constraining effects at higher percentile levels, particularly at extremely high levels (see Fig 5 and Table 13).

**Table 12:** Effect sizes of NCA.

Construct	Taste Inference				Product-related Cognitive Engagement			
	CE-FDH	p-value	CR-FDH	p-value	CE-FDH	p-value	CR-FDH	p-value
Perceived Naturalness	0.097	0.000	0.084	0.013	0.079	0.014	0.071	0.033
Perceived Credibility	0.069	0.068	0.156	0.000	0.227	0.000	0.206	0.000
Age	0.000	1.000	0.000	1.000	0.000	1.000	0.000	1.000
Taste Inference					0.414	0.000	0.379	0.000
Age × Taste Inference					0.000	1.000	0.000	1.000

**Table 13:** Bottleneck table of product-related cognitive engagement (percentages).

Dependent variable: Product-related Cognitive Engagement								
Percentile (%)	CE-FDH				CR-FDH			
	Perceived Naturalness	Perceived Credibility	Taste Inference	Age × Taste Inference	Perceived Naturalness	Perceived Credibility	Taste Inference	Age × Taste Inference
0	NN	NN	NN	NN	NN	NN	NN	NN
10	NN	NN	8.5%	NN	NN	NN	6.3%	NN
20	NN	0.0%	27.8%	NN	NN	NN	14.2%	NN
30	NN	0.0%	31.6%	NN	NN	0.9%	22.1%	NN
40	NN	23.7%	35.7%	NN	NN	9.1%	30.0%	NN
50	NN	23.7%	43.8%	NN	NN	17.2%	37.9%	NN
60	3.4%	23.7%	47.5%	NN	NN	25.4%	45.8%	NN
70	3.4%	27.9%	48.1%	NN	NN	33.6%	53.7%	NN
80	3.4%	27.9%	60.0%	NN	14.6%	41.7%	61.6%	NN
90	26.7%	60.6%	69.8%	NN	32.8%	49.9%	69.5%	NN
100	63.6%	61.5%	84.1%	NN	50.9%	58.1%	77.4%	NN

NN means not necessary, and 0.0% represents the minimum value.

Overall, the NCA results align with the PLS-SEM findings, highlighting taste inference, credibility, and perceived naturalness as critical bottleneck variables. Among them, taste inference and credibility play central roles in facilitating higher levels of product-related cognitive engagement, while age did not emerge as a notable necessary condition. Additionally, perceived naturalness and credibility were examined as necessary conditions for taste inference; perceived naturalness showed the stronger and more consistent effect (CE-FDH = 0.097, p < 0.001), while credibility’s necessity was method-dependent (CR-FDH = 0.156, p < 0.001; CE-FDH = 0.069, p = 0.068, see Tables 12 and 14). It should be noted that the NCA results reported here are exploratory and context-specific. The identified threshold levels reflect the specific sample distribution and should not be interpreted as universal or natural cutoffs applicable to other populations or platform settings. They serve as supplementary evidence for hierarchical processing assumptions within the Chinese e-commerce context.

**Table 14:** Bottleneck table of taste inference (percentages).

Dependent variable: Taste Inference				
Percentile (%)	CE-FDH		CR-FDH	
	Perceived Naturalness	Perceived Credibility	Perceived Naturalness	Perceived Credibility
0	NN	NN	NN	NN
10	NN	0.0%	NN	NN
20	NN	0.0%	NN	NN
30	NN	0.0%	NN	NN
40	NN	0.0%	NN	NN
50	NN	0.0%	NN	NN
60	NN	0.0%	NN	12.3%
70	NN	0.0%	NN	24.8%
80	22.4%	0.0%	3.3%	37.4%
90	23.2%	44.3%	41.8%	49.9%
100	90.5%	72.9%	80.3%	62.4%

NN means not necessary, and 0.0% represents the minimum value.

#### Integration of analytical perspectives.

The convergence and divergence across the three analytical methods provide a nuanced diagnostic of the cognitive processing model. When PLS-SEM and NCA converge on the same predictor (e.g., taste inference as sufficient in PLS-SEM, β = 0.422, p < 0.001, and necessary in NCA, CE-FDH = 0.414, p < 0.001), this indicates a critical bottleneck variable that is both a strong linear contributor and a minimum requirement—suggesting that interventions targeting this construct will have high leverage.

When PLS-SEM shows significance but NCA does not (e.g., PN → PCE: β = 0.092, p < 0.05; NCA CE-FDH = 0.079, p = 0.014), the predictor operates as a facilitator that enhances outcomes when present but does not severely constrain outcomes when absent. Furthermore, MGA-qualified paths (e.g., PN → PCE significant only for natural images, β = 0.285, p = 0.002 vs. β = 0.026, p = 0.636 for lifestyle images) indicate context-dependent sufficiency, where the general necessity identified by NCA is activated only under specific boundary conditions.

This tripartite analytical architecture thereby disentangles three distinct causal questions: the average linear contribution of predictors (sufficiency), the contextual boundaries of these contributions (moderation), and the minimum required conditions for high outcome states (necessity).

## Discussion and conclusion

This study examined how perceived naturalness, credibility, and taste inference jointly shape product-related cognitive engagement in e-commerce contexts. Drawing on analyses of psychological pathways, contextual moderators, and necessary conditions, the study identified key processes through which visual and textual cues influence consumer cognition in digital presentations of natural foods. The integration of PLS-SEM, MGA, and NCA enabled assessment of direct and indirect effects, contextual boundaries, and minimum required conditions.

The results indicate that perceived naturalness operates through multiple pathways: a direct effect on cognitive engagement, parallel indirect effects via credibility and taste inference, and a sequential chain in which credibility enables taste inference that subsequently sustains elaboration. Natural-scene imagery and consumption-oriented message frames are associated with stronger central processing and enhanced authenticity attribution. Content-oriented platforms strengthen the relationship between credibility and cognitive engagement. Findings from NCA complement the sufficiency-based results by identifying taste inference as the strongest necessary condition for high cognitive engagement, followed by credibility; perceived naturalness showed a weaker but significant necessity effect. In addition, age emerged as a boundary condition, weakening the link between taste inference and cognitive engagement, particularly on content-oriented platforms where elaborative processing is normative.

Overall, the findings clarify how perceptions of naturalness, credibility, and imagined taste jointly contribute to cognitive engagement in digital marketing contexts for natural foods. By combining analyses of psychological mechanisms, contextual factors (platform type and visual–verbal cues), individual differences (age), and necessary conditions, the study provides an integrated account of authenticity-related cognitive processing. These results have implications for the design of visual–verbal information and platform strategies aimed at fostering consumer trust, cognitive engagement, and value co-creation.

### Theoretical contributions

This study offers several key contributions to the fields of digital food marketing and consumer cognition. First, we outline a multifaceted network of pathways through which perceptual cues translate into inferential cognition prior to consumption. While prior research has extensively explored food-related information processing in social commerce contexts [74], the specific network of pathways through which perceptual cues translate into inferential cognition during the pre-consumption stage remains less understood. Our findings extend this line of inquiry by demonstrating that perceived naturalness does not merely trigger direct outcomes, but operates through a multifaceted combination of parallel and sequential mechanisms involving credibility and taste inference.

Second, this research refines the role of perceived naturalness, positioning it as an antecedent of authenticity-related attribution rather than a mere aesthetic signal [60]. Natural imagery serves as a cue of “origin-based realness,” facilitating deeper exploratory cognition through credibility and taste inference. This pattern suggests that authenticity-related peripheral cues can be associated with more effortful processing, offering an interpretation consistent with the ELM in food evaluation contexts. In this sense, the findings complement existing research on visual and verbal food cues by highlighting the role of authenticity-related perceptions in shaping processing depth [19,75].

Third, by linking sensory cue processing to cognitive engagement (e.g., information-seeking and elaborative thinking), we bridge the gap between sensory marketing and value co-creation literature [74,76]. The multi-group analysis shows that consumption-oriented textual frames strengthen the association between credibility and taste inference, particularly in content-oriented platforms. This finding suggests that sensory expectations may be connected to early-stage cognitive forms of value co-creation, extending prior work that has focused primarily on aesthetic appeal or surface-level attention outcomes [63].

Finally, our tripartite analytical approach (PLS-SEM, MGA, and NCA) provides a dynamic perspective on authenticity attribution. Results from NCA complement the PLS-SEM findings by identifying taste inference as the strongest necessary condition for high cognitive engagement, followed by credibility; perceived naturalness showed a weaker but significant necessity effect. These patterns suggest that while all three constructs facilitate engagement on average (sufficiency), taste inference is the most critical minimum requirement for achieving high engagement states. This dynamic perspective differs from approaches that conceptualize external food cues as fixed heuristics, suggesting instead that their influence depends on consumers’ authenticity-related interpretations [47,62,77].

In addition, the findings highlight the role of contextual factors in shaping anticipatory food processing. Nature-oriented images are more strongly associated with perceived naturalness than lifestyle scenes; content-oriented platforms are linked to deeper inferential processing; and consumption-oriented textual frames facilitate credibility-based taste simulation. Together, these patterns indicate that digital food environments structure authenticity perception and sensory anticipation in context-dependent ways [32,78,79].

Overall, the results describe a layered psychological process underlying pre-consumption food evaluation. Perceived naturalness provides an initial cue of realness that can operate through multiple psychological channels; credibility supports trust-based inference; and taste inference is closely associated with deeper cognitive engagement. Age-related differences further delineate processing boundaries, with younger consumers showing stronger tendencies toward taste simulation and elaborative engagement, and older consumers exhibiting attenuated translation of taste inference into sustained cognitive effort, particularly in content-oriented platform contexts where elaborative processing is normative [80–82]. These findings offer an integrated view of how authenticity-related perceptions, contextual factors, and individual differences jointly shape anticipatory sensory cognition in digital food contexts.

### Managerial implications

The findings offer practical implications for the design of digital food environments, particularly for e-commerce platforms where pre-consumption expectations are critical. These recommendations are grounded in the observed psychological pathways and boundary conditions identified through PLS-SEM, MGA, and NCA.

First, natural visual cues should be deployed to prioritize authenticity attribution. Since natural-scene imagery significantly strengthens the path from perceived naturalness to credibility, practitioners should utilize origin landscapes or ecological farming scenes. Such visuals act as “attributional gatekeepers”, fostering the trust necessary for further consumer engagement. To maintain this signal, overly stylized or artificial decorations should be avoided as they may dilute the perceived realness of the food product.

Second, content strategies must be tailored to the specific platform environment. The MGA results indicate that content-oriented platforms facilitate deeper inferential processing. Consequently, in these environments, marketers should leverage immersive narratives and consumption-oriented frames to enhance experiential engagement. In contrast, for transaction-oriented settings, the focus should shift toward combining restrained natural imagery with explicit credibility indicators (e.g., certifications or traceability labels) to streamline the trust-based evaluation process.

Third, sensory-focused information should be prioritized as a necessary foundation for engagement. Drawing on the NCA finding that taste inference is the strongest necessary condition for high cognitive engagement, designers must ensure that sensory cues—such as vivid depictions of harvesting or origin processes—are sufficiently present. Without effectively stimulating the consumer’s “imagined taste”, even high levels of perceived naturalness may fail to trigger deep cognitive involvement, such as information-seeking or elaborative thinking.

Fourth, communication strategies should account for consumer heterogeneity, particularly age-related differences. Since age weakens the link between taste inference and cognitive engagement, a segmented approach is required. For younger consumers, marketers can utilize rich flavor descriptions and process demonstrations to stimulate anticipatory taste. For older consumers, the emphasis should be placed on grounding taste-related information in familiar reference points and traditional production methods to reduce cognitive effort and enhance evaluative confidence.

By strategically navigating these psychological mechanisms and contextual boundaries, e-commerce practitioners can foster more robust cognitive engagement, moving beyond simple visual appeal to create deep-seated consumer trust and interest.

### Limitations and future research

Several limitations suggest avenues for future work. First, external validity may be constrained by both the experimental setting and the scope of generalizability. The study employed controlled stimulus presentations that, while ensuring internal validity, may not fully capture the complexity of real-world e-commerce interfaces. Actual digital food environments involve dynamic social cues (e.g., user reviews, live interactions), goal-directed browsing behaviors, and competing attention demands that could shape cognitive and affective processing differently from the laboratory context. Moreover, although online sample services offer efficient recruitment, they face emerging risks from AI-simulated respondents and automated bots that can compromise data integrity. While we implemented rigorous attention checks and platform-level screening to mitigate these threats, the increasing sophistication of synthetic agents remains a persistent challenge for digital behavioral research. Furthermore, our data were collected from Chinese e-commerce users, and the sample is characterized by young (80.3% aged 18–35), well-educated (83.1% with bachelor’s and/or master’s degrees), digitally engaged consumers. While this aligns with the target demographic of natural food marketing on Chinese platforms, caution is warranted when extending these findings to other cultural contexts or consumer segments. The Chinese e-commerce ecosystem exhibits distinctive features—such as integrated social shopping and platform-specific trust mechanisms—that may moderate the effects of visual and textual cues differently than in Western markets. Future research could combine experimental designs with eye-tracking or platform-level behavioral data to validate the robustness of laboratory findings in naturalistic browsing settings, and should replicate and extend our model in diverse geographical and platform settings to assess cross-cultural robustness.

Second, stimulus specificity limits the generalizability of the findings. The present study deliberately focuses on message-level visual and textual cues for natural foods and the inferential mechanisms they activate, rather than on individual sensory traits. The stimulus set was restricted to natural food categories and specific types of visual scenes (natural landscapes vs. lifestyle contexts) and textual frames (consumption-oriented vs. production-oriented). Future research could expand the stimulus space to include processed foods, sensory resonance cues, brand-signal consistency, novelty cues, or AI-generated and synthetic visuals to clarify how multisensory information jointly shapes anticipatory food experiences [50,83]. Additionally, integrating consumer-level differences relevant to food perception—such as imagery vividness, sensory sensitivity, food neophobia, or embodied simulation tendencies—could examine how these factors moderate or amplify the proposed pathway from perceived naturalness to credibility and taste-related reasoning.

Third, the cross-sectional design precludes causal claims about long-term behavioral engagement. The present study captures cognitive responses at a single point in time and cannot address how short-term cognitive and sensory inferences evolve into sustained consumer-brand co-creation relationships. Longitudinal designs could track emotional engagement, content sharing, and user-generated contributions over time, illuminating how anticipatory judgments translate into sustained behavioral patterns.

Fourth, the measurement of cognitive engagement relies on self-reported indicators rather than behavioral proxies. While the study captures consumers’ subjective reports of information-seeking motivation and elaborative thinking, actual behavioral measures (e.g., click-through rates, dwell time, information search depth) could provide complementary validation of the proposed pathway. All measurement items were positively valenced, which may inflate inter-construct correlations through acquiescence bias. While we implemented procedural and statistical safeguards (randomized item order, Harman’s test, full collinearity assessment), the absence of reverse-scored items remains a limitation. Future research should incorporate both behavioral metrics and mixed-valence item sets to strengthen the empirical foundation and rule out method bias.

## Supporting information

S1 File Sensitivity analysis: Mode A versus Mode B estimation. The file contains two tables: (1) path coefficient comparison across Mode A and Mode B, including differences, p-values, and hypothesis support; (2) model fit and explained variance comparison.
(XLSX)

S2 File Survey data. The dataset contains individual-level responses from 320 participants in CSV format. Variable definitions: PN (perceived naturalness), PC (perceived credibility), TI (taste inference), PCE (product-related cognitive engagement), image_check/title_check/platform_check (manipulation checks), platform_familiarity/cover_liking (manipulation check controls), image_type/title_type/platform_type (experimental conditions, 0/1 coding).
(CSV)
